# Chlorantraniliprole Formulation Poisoning: A Signal for Cardiac Conduction Abnormalities

**DOI:** 10.1002/ccr3.73632

**Published:** 2026-09-24

**Authors:** Prashim Sharma, Nishan Subedi, Kanchan Thulung Rai, Alisha Adhikari

**Affiliations:** ^1^ Birat Medical College Teaching Hospital Morang Nepal; ^2^ Department of Medicine Birat Medical College Teaching Hospital Morang Nepal

**Keywords:** atrioventricular block, cardiac conduction, chlorantraniliprole, insecticide poisoning, ryanodine receptor

## Abstract

Chlorantraniliprole poisoning is rarely reported in humans. A 25‐year‐old man developed sinus bradycardia and first‐degree atrioventricular block after ingesting a chlorantraniliprole 18.5% suspension concentrate formulation. Laboratory investigations were unremarkable, and ECG abnormalities resolved within 72 h with supportive care. ECG monitoring may be warranted in similar presentations.

## Key Clinical Message

Ingestion of a chlorantraniliprole 18.5% suspension concentrate formulation may be associated with transient atrioventricular conduction abnormalities. Although causality cannot be established, ECG monitoring should be considered in similar presentations until additional human data become available.

## 1 Introduction

Pesticide poisoning remains a significant cause of morbidity in agricultural regions, particularly following intentional ingestion. While organophosphate and carbamate toxicities are well characterized, human data on newer pesticide classes remain limited.

Chlorantraniliprole is an anthranilic diamide insecticide that acts on insect ryanodine receptors, causing dysregulated intracellular calcium release and neuromuscular paralysis [1]. Chlorantraniliprole has high selectivity for insect ryanodine receptors over mammalian receptors, contributing to a favorable safety profile in standard toxicological evaluations [2, 3]. However, the selectivity is not absolute, and human poisoning data remain rare.

A prior case described Mobitz Type I AV block requiring temporary pacing following chlorantraniliprole ingestion [4]. We report a case of transient first‐degree AV block following ingestion of a chlorantraniliprole 18.5% suspension concentrate formulation, and discuss the difficulty of attributing observed effects to the active ingredient versus formulation adjuvants.

## 2 Case History and Examination

**FIGURE 1:**
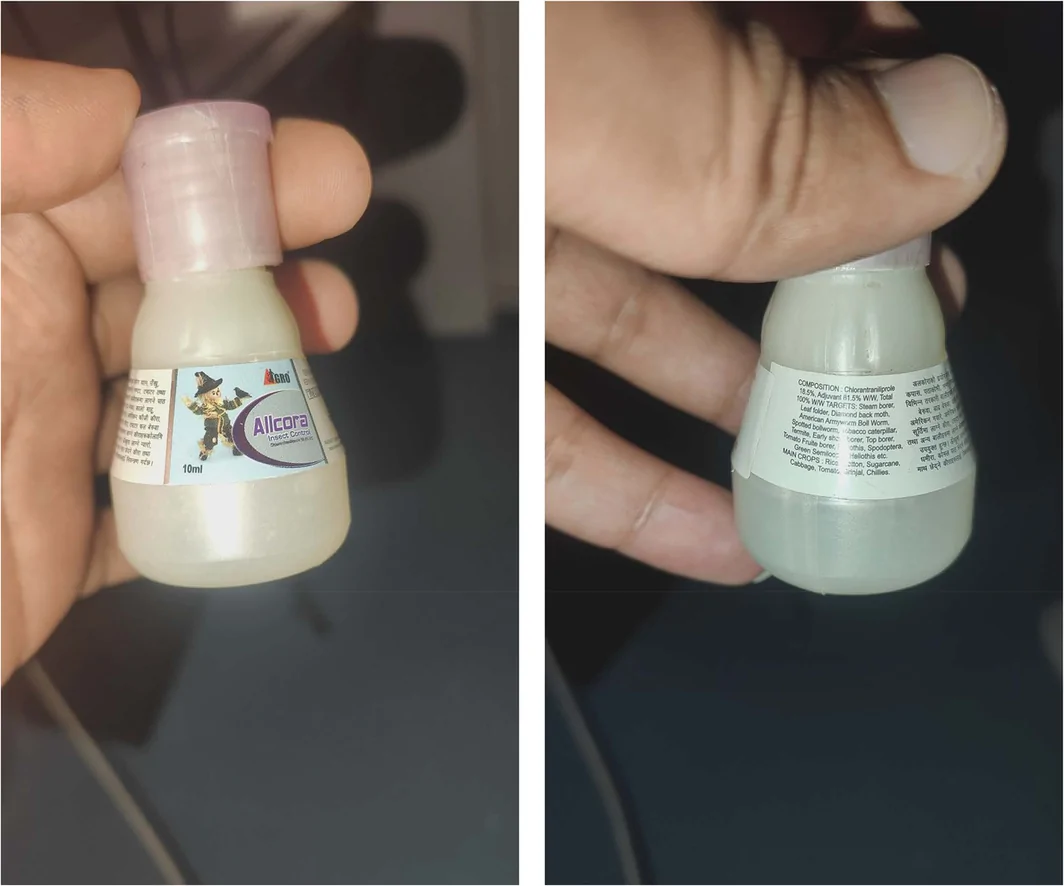
Commercial formulation of chlorantraniliprole 18.5% SC (Allcora) associated with the reported ingestion.

A previously healthy 25‐year‐old man presented to the emergency department approximately 2 h after deliberate ingestion of approximately 10 mL of ALLCORA (chlorantraniliprole 18.5% w/w; adjuvants 81.5% w/w). The product is shown in Figure 1. He developed vomiting within 30 min of ingestion. No chest pain, syncope, palpitations, seizures, or respiratory symptoms were reported. There was no history of convulsions or muscle weakness before or after the ingestion. Co‐ingestants could not be definitively excluded as comprehensive toxicological screening was unavailable. Physical examination findings are summarized in Table 1.

**TABLE 1:** Clinical examination findings at presentation.

Parameter	Findings
Blood pressure	110/70 mmHg
Pulse rate	58 beats/min, regular
Respiratory rate	16 breaths/min
SpO_2_	98% on room air
Temperature	37.1°C
ECG	Sinus bradycardia with first‐degree AV block (PR 220 ms), narrow QRS, QTc within normal limits, no ischaemic changes

## 3 Differential Diagnosis, Investigations and Treatment

Differential diagnoses included organophosphate poisoning, carbamate poisoning, mixed pesticide ingestion, and toxicity related to chlorantraniliprole formulation ingestion. Normal cholinesterase levels and absence of cholinergic features made organophosphate poisoning unlikely.

### 3.1 Investigations

ECG on arrival (approximately 2 h post‐ingestion) showed sinus bradycardia with first‐degree AV block (PR interval 220 ms), narrow QRS complexes, normal QTc interval, and no ischaemic changes. Full blood count, renal and liver function tests, serum electrolytes, and thyroid profile were within normal limits. Troponin I, CK‐MB, and serum cholinesterase levels were also normal.

Clinical and biochemical findings were not consistent with organophosphate toxicity: serum cholinesterase was normal and the clinical phenotype (isolated bradycardia and AV block without miosis, hypersalivation, bronchospasm, or fasciculations) did not support a cholinergic toxidrome.

### 3.2 Management

Gastric lavage was performed approximately 2 h after ingestion. Although current evidence‐based guidelines generally do not recommend lavage beyond 60 min post‐ingestion, the decision was made on clinical grounds given the deliberate nature of the ingestion, the uncertain bioavailability of a suspension concentrate formulation, and the potential for systemic toxicity from unknown adjuvant components. The authors acknowledge that this approach is not supported by current routine poisoning management guidelines. Atropine was administered empirically during early assessment as organophosphate toxicity had not yet been excluded; there was no clinically apparent response, and it was not used diagnostically. Supportive care included intravenous fluids and antiemetics. Continuous cardiac monitoring was maintained for 72 h.

### 3.3 Estimated Exposure and Toxicological Context

The reported ingestion volume of approximately 10 mL corresponds to an estimated formulation mass of approximately 10–12 g, assuming a density of 1–1.2 g/mL (a range typical for suspension concentrates; the actual product density was not available). Based on the labeled active ingredient concentration of 18.5% w/w, this represents an estimated ingested chlorantraniliprole content of approximately 1.85 g. This figure represents estimated ingested dose based on reported volume and labeled concentration; systemic absorbed dose is unknown and cannot be inferred, as bioavailability data for this formulation are not available.

In animal studies, chlorantraniliprole demonstrates very high oral LD^50^ values (> 2000 mg/kg in rodents), indicating a wide margin in standard safety evaluations [3]. Direct extrapolation of these values to human toxicity at lower doses is not valid.

### 3.4 Causality Assessment (WHO‐UMC)

The association between formulation ingestion and the observed conduction abnormality is classified as possible using the WHO‐UMC system, based on the following:

- Temporal relationship between ingestion and onset of ECG abnormalities
- Exclusion of common alternative causes (normal electrolytes, thyroid function, cardiac biomarkers, and cholinesterase; clinical phenotype inconsistent with organophosphate toxicity)
- Positive dechallenge (resolution of ECG changes with clinical recovery)
- Theoretical biological plausibility via ryanodine receptor‐mediated calcium dysregulation, noting that this mechanism remains speculative in humans
- Absence of rechallenge

Importantly, 81.5% of the ingested formulation comprised adjuvants of unknown composition. Causal attribution cannot be assigned to the active ingredient alone (see Discussion).

## 4 Results (Outcome and Follow‐up)

No further atropine therapy, vasopressors, or temporary pacing was required. The patient remained hemodynamically stable throughout hospitalization. Serial ECG monitoring demonstrated gradual resolution of PR prolongation, with return to normal sinus rhythm within 72 h. He was discharged in stable condition without residual symptoms.

## 5 Discussion

### 5.1 Known Mechanism of Chlorantraniliprole in Insects

Chlorantraniliprole acts selectively on insect ryanodine receptors, causing sustained intracellular calcium release, neuromuscular paralysis, and death [1]. This mechanism is well characterized in target species and underlies its efficacy as an insecticide.

### 5.2 Mammalian Ryanodine Receptor Uncertainty

Mammalian ryanodine receptors—particularly RyR2, the predominant cardiac isoform—are substantially less sensitive to chlorantraniliprole than insect receptors [2, 3]. However, the selectivity is not absolute. Cardiac RyR2 is known to regulate intracellular calcium release during excitation–contraction coupling and contributes to sinoatrial automaticity in mammals [5, 6]. Whether chlorantraniliprole at clinically relevant ingested doses can meaningfully interact with human RyR2 is not established. Any mechanistic inference from this case must therefore be regarded as speculative.

### 5.3 Formulation Composition as the Primary Interpretive Axis

The formulation ingested in this case contained 81.5% adjuvants by weight, whose composition was not available. This represents the dominant source of toxicological uncertainty. Pesticide formulations frequently contain surfactants, emulsifiers, and organic solvents that are capable of independently producing gastrointestinal and cardiovascular effects. Cardiovascular effects have been described in the context of pesticide formulation poisoning, with adjuvants proposed as contributing factors in some cases. Therefore, the observed sinus bradycardia and first‐degree AV block in this case may reflect toxicity of the adjuvant components, the active ingredient, or an interaction between both. This case is more accurately characterized as a formulation‐associated toxicological event than as chlorantraniliprole‐specific cardiotoxicity.

### 5.4 Human Evidence: This Case and the Prior Report

A previously published case described Mobitz Type I AV block requiring temporary pacing following chlorantraniliprole ingestion [4]. The present case demonstrates a milder, reversible conduction abnormality (first‐degree AV block with spontaneous resolution within 72 h) without requirement for intervention. Taken together, these two cases suggest a possible range of formulation‐associated AV conduction abnormalities, from transient PR prolongation to higher‐degree block, potentially influenced by ingested volume, formulation composition, and individual susceptibility. Quantitative dose comparison between the two cases is not possible given the differing reported volumes and absent pharmacokinetic data.

Experimental data in honey bees demonstrate chlorantraniliprole‐associated cardiac rhythm disturbances including bradycardia and cardiac arrest in isolated bee hearts [7]. Extrapolation of these findings to human electrophysiology is substantially limited by phylogenetic and physiological differences, and this evidence is cited only to illustrate that insect cardiac tissue can be affected; it does not validate a human mechanistic inference.

### 5.5 Integrated Interpretation

The net interpretation of this case is that ingestion of a chlorantraniliprole 18.5% suspension concentrate formulation was associated with a transient, reversible AV conduction abnormality in a previously healthy young adult. Whether this reflects activity of the active ingredient at human cardiac ryanodine receptors, adjuvant‐mediated cardiovascular toxicity, or both cannot be determined from a single case. The association is classified as possible under the WHO‐UMC system, and this classification should be understood as a signal for further observation rather than a confirmed mechanistic attribution.

### 5.6 Limitations

- Single case; causality cannot be confirmed and generalization is not possible
- Ingested dose expressed as estimated formulation mass and active ingredient content based on labeled concentration and assumed density (1–1.2 g/mL); systemic absorbed dose is unknown
- Adjuvant composition unknown; independent cardiovascular contribution of adjuvants cannot be excluded and may be substantial
- Serum chlorantraniliprole levels were not measured
- Co‐ingestants not definitively excluded
- Gastric lavage performed outside standard guideline recommendations; this may have modified toxin absorption and altered the natural clinical course
- Organophosphate toxicity excluded on clinical and biochemical grounds (normal cholinesterase, absence of cholinergic signs); atropine administered empirically during early assessment but not used diagnostically
- Manufacturer details for ALLCORA were not available at time of presentation

## 6 Conclusion

Ingestion of a chlorantraniliprole 18.5% suspension concentrate formulation was associated with transient sinus bradycardia and first‐degree AV block in a previously healthy young adult, with spontaneous resolution within 72 h. The association is classified as possible using the WHO‐UMC system. The observed conduction abnormality may reflect activity of the active ingredient, formulation adjuvants, or both; definitive attribution is not possible. This case is best understood as a formulation‐associated toxicological signal rather than confirmed chlorantraniliprole cardiotoxicity. ECG monitoring may be considered in similar presentations, particularly when cardiovascular symptoms or abnormal vital signs are present, pending further case accumulation.

## Author Contributions

**Prashim Sharma:** conceptualization, data curation, methodology, visualization, writing – original draft. **Nishan Subedi:** project administration, resources, investigation, writing – review and editing. **Kanchan Thulung Rai:** writing – review and editing. **Alisha Adhikari:** formal analysis, validation, writing – review and editing.

## Funding

The authors have nothing to report.

## Ethics Statement

Ethics committee review was not required according to institutional policy.

## Consent

Written informed consent was obtained from the patient for publication of this case report.

## Conflicts of Interest

The authors declare no conflicts of interest.

## Data Availability

The data that support the findings of this study are available from the corresponding author upon reasonable request.
